# Brain metastases-derived extracellular vesicles induce binding and aggregation of low-density lipoprotein

**DOI:** 10.1186/s12951-020-00722-2

**Published:** 2020-11-07

**Authors:** Sara Busatto, Yubo Yang, Sierra A. Walker, Irina Davidovich, Wan-Hsin Lin, Laura Lewis-Tuffin, Panagiotis Z. Anastasiadis, Jann Sarkaria, Yeshayahu Talmon, Gregory Wurtz, Joy Wolfram

**Affiliations:** 1 Department of Biochemistry and Molecular Biology, Department of Physiology and Biomedical Engineering, Department of Transplantation, Mayo Clinic, Jacksonville, FL 32224 USA; 2 Vascular Biology Program, Boston Children’s Hospital, Boston, MA USA; 3 Department of Surgery, Boston Children’s Hospital and Harvard Medical School, Boston, MA USA; 4 Department of Cancer Biology, Mayo Clinic Comprehensive Cancer, Center, Mayo Clinic, Jacksonville, FL USA; 5 Department of Chemical Engineering and the Russell Berrie Nanotechnology Institute (RBNI), Technion-Israel Institute of Technology, 3200003 Haifa, Israel; 6 Department of Radiation Oncology, Mayo Clinic, Rochester, MN 55902 USA; 7 Department of Physics, University of North Florida, Jacksonville, FL 32224 USA

**Keywords:** Extracellular vesicles, Lipoproteins, Brain metastasis, Macrophages, Pre-metastatic niche

## Abstract

**Background:**

Cancer cell-derived extracellular vesicles (EVs) have previously been shown to contribute to pre-metastatic niche formation. Specifically, aggressive tumors secrete pro-metastatic EVs that travel in the circulation to distant organs to modulate the microenvironment for future metastatic spread. Previous studies have focused on the interface between pro-metastatic EVs and epithelial/endothelial cells in the pre-metastatic niche. However, EV interactions with circulating components such as low-density lipoprotein (LDL) have been overlooked.

**Results:**

This study demonstrates that EVs derived from brain metastases cells (Br-EVs) and corresponding regular cancer cells (Reg-EVs) display different interactions with LDL. Specifically, Br-EVs trigger LDL aggregation, and the presence of LDL accelerates Br-EV uptake by monocytes, which are key components in the brain metastatic niche.

**Conclusions:**

Collectively, these data are the first to demonstrate that pro-metastatic EVs display distinct interactions with LDL, which impacts monocyte internalization of EVs.

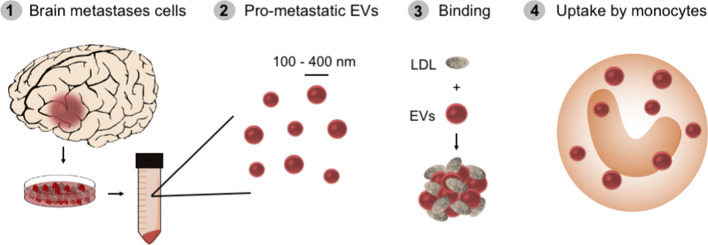

## Background

Metastasis accounts for up to 90% of all cancer-related deaths [1, 2]. However, many aspects of the metastatic process are poorly understood, limiting the development of diagnostic and therapeutic strategies to prevent metastatic spread. Recently, it was shown that cancer cell-derived extracellular vesicles (EVs) contribute to pre-metastatic niche formation [3, 4]. EVs are cell-secreted nanoparticles surrounded by a lipid bilayer enclosing bioactive cargo (proteins, carbohydrates, lipids, and nucleic acids) involved in cell communication in both physiological and pathological conditions [5–8]. Aggressive primary tumors have been found to secrete pro-metastatic EVs that travel within the circulation to distant organs, causing modulation of the microenvironment for future metastatic spread [3, 4].

Animal studies have demonstrated that pretreatment with pro-metastatic EVs prior to cancer cell injection can substantially increase the formation of subsequent metastatic lesions [4, 9, 10]. Evidence suggests that EV-mediated metastatic organotropism involves integrin interactions with tissue-specific epithelial, endothelial, or resident immune cells [4, 11]. Cancer cell-derived EVs have also been shown to carry microRNAs (miRNAs) that disrupt the integrity of endothelial barriers in distant organs [12, 13]. However, minimal focus has been placed on pro-metastatic EV interactions with circulating components, such as low-density lipoprotein (LDL), which has been associated with cancer progression [14–16]. Specifically, LDL is known to play a role in acidic tumor microenvironments, supporting cancer growth and metastasis [17, 18].

In this study, interactions between LDL and EVs derived from cancer types that are prone to metastasize to the brain (breast cancer and melanoma [19–21]) have been explored. Specifically, comparative analyses were performed between regular EVs (Reg-EVs) and brain metastases EVs (Br-EVs) from breast cancer and melanoma cells, focusing on LDL binding, aggregation, and subsequent effects on brain endothelial cells and monocytes. Exploring interactions that occur between pro-metastatic EVs and LDL has the potential to shed light on unknown aspects of the metastatic process.

## Results

### Reg-EVs and Br-EV display similar physical and biochemical characteristics

**Fig. 1:**
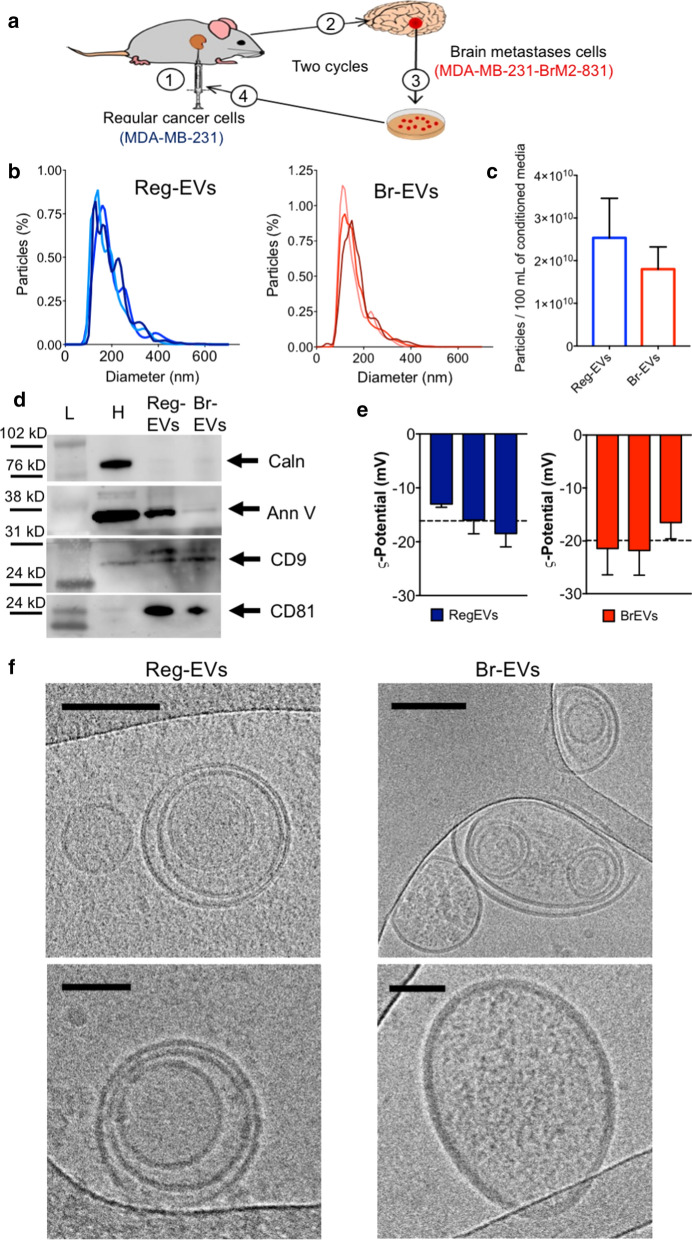
Characterization of extracellular vesicles (EVs) derived from regular (Reg) and brain metastases (Br) breast cancer cells. The conditioned media of regular human MDA-MB-231 breast cancer cells and a brain metastases variant of MDA-MB-231 cells (MDA-MB-231-BrM2-831) were processed by tangential flow filtration to obtain Reg-EVs and Br-EVs, respectively**. a** Schematic of in vivo selection cycles to obtain a brain metastases cell line: (1) intracardiac injection of cells, (2) development of brain metastases, (3) excision of brain metastases and in vitro culture, and (4) intracardiac injection of cultured cells. **b** Size distribution profiles (10 nmincrements) measured by nanoparticle tracking analysis (NTA). Data are shown as biological triplicates. **c** Particles isolated from 100 mL of conditioned cell culture media. **d** Protein markers of EVs (annexin V, cluster of differentiation (CD)9, and CD81) and intracellular contaminants (calnexin) obtained by Western blot. Arrows represent the expected protein band. **e** Zeta potential measured by laser Doppler micro-electrophoresis. Data are presented as mean ± SD of three technical replicates. Dashed line represents the mean value of three biological replicates. **f** Representative cryogenic transmission electron microscopy images. Scale bar, 100 nm (upper panels) and 50 nm (lower panels). *Ann V* annexin V, *Caln* calnexin, *H* *cell homogenate*; *L* protein ladder

Breast cancer cell-derived Reg-EVs were isolated from the conditioned medium of human MDA-MB-231 breast cancer cells. Corresponding Br-EVs were obtained from the conditioned medium of a previously characterized brain-topic variant of MDA-MB-231 cells (MDA-MB-231-BrM2-831), which was derived from successive implantation, resection, and re-implantation of brain-seeking MDA-MB-231 cells in a mouse model (Fig. 1a) [22]. The gene expression profile of the brain-tropic cell line correlates with that of breast cancer brain metastases cells isolated from patients [22], indicating the presence of clinically relevant metastatic features despite being obtained through in vivo selection cycles in an animal model. Previous comparisons of the pro-metastatic potential of intravenously injected MDA-MB-231 Reg-EVs and Br-EVs have revealed that only the latter increases the prevalence and size of metastatic brain lesions upon subsequent intracardiac injection of cancer cells [9], demonstrating that these EVs retain the non-metastatic and metastatic features of the originating cells. The common origin of the regular and brain metastases MDA-MB-231 cell variants provides a suitable model system for studying brain metastasis, as differences in these cell lines are largely attributed to the metastatic process [9]. Therefore, this study has primarily relied on comparisons between Reg-EVs and Br-EVs obtained from these MDA-MB-231 cell variants. Additional studies were performed on EVs isolated from the conditioned medium of primary human A375 melanoma cells and human M12 melanoma brain metastases cells of unrelated origin (Additional file 1: Fig. S1a).

Tangential flow filtration (TFF) [23, 24] was used for efficient, pure, and consistent isolation of EVs, which were characterized according to guidelines of the International Society of Extracellular Vesicles [25]. Nanoparticle tracking analysis (NTA) demonstrated that EVs derived from breast cancer (Fig. 1b and c) and melanoma (Additional file 1: Fig. S1b and c) cell lines had a concentration of 1.8–3.2 × 10^10^/100 mL of conditioned media (~ 250 EVs/seeded cell in 48 h) and a narrow multimodal size distribution with the majority of EVs being in the 100–200 nm size range. All EVs were enriched in annexin V and the tetraspanins cluster of differentiation (CD)9 and/or CD81, which are known EV markers [25] (Fig. 1d and Additional file 1: Fig. S1d). The EVs were also negative for calnexin, an endoplasmic reticulum protein, which is typically used as a marker of intracellular vesicle contaminants (Fig. 1c and Additional file 1: Fig. S1c) [25]. The EVs displayed a negative zeta potential (Fig. 1d), which is expected [26]. Morphological analysis with atomic force microscopy (AFM) (Additional file 1: Fig. S1d) and cryogenic transmission electron microscopy (cryo-TEM) (Fig. 1e) showed the presence of round-shaped nanosized particles surrounded by negligible excess material. Cryo-TEM images demonstrated that EVs consisted of unilamellar and multilamellar phospholipid bilayer structures (Fig. 1e), which is consistent with other studies [24, 27–30]. These results also indicate that Reg-EVs and Br-EVs have similar physical and biochemical characteristics.

### Reg-EVs and Br-EV display similar interaction with brain endothelial cells

**Fig. 2:**
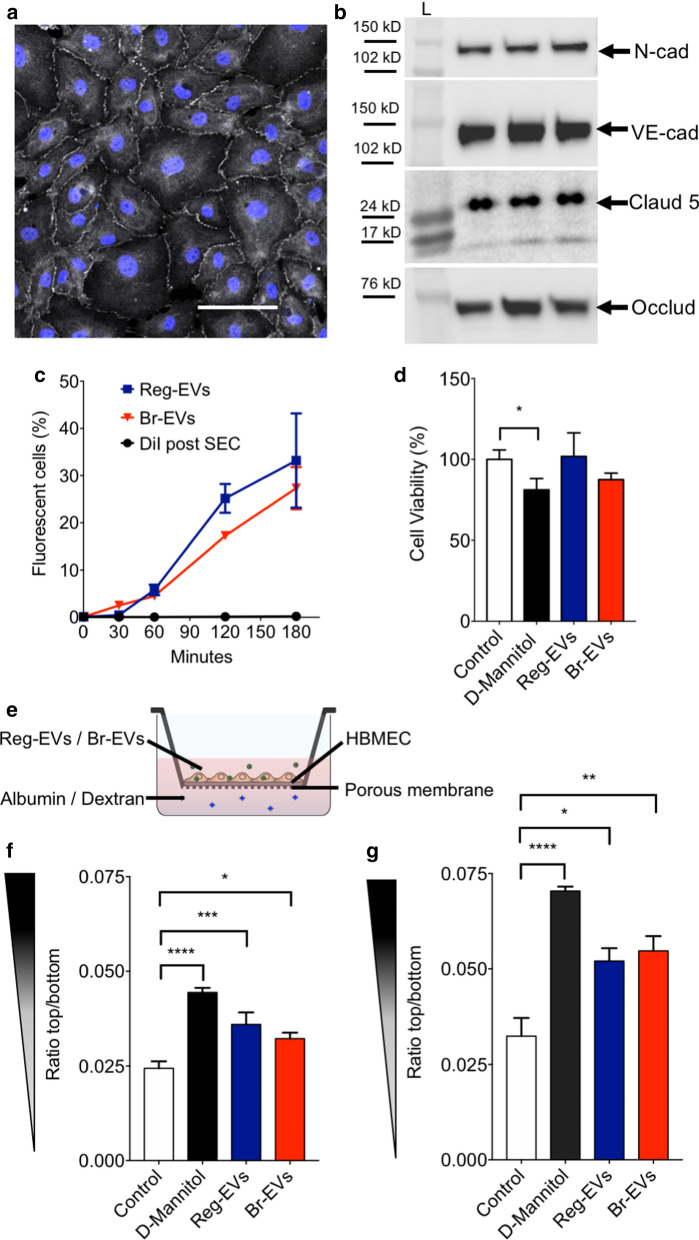
Effects of breast cancer cell-derived Reg-EVs and Br-EVs on brain endothelial cells. Reg EVs were derived from MDA-MB-231 cells and Br-EVs from MDA-MB-231-BrM2-831 cells. **a** Confocal analysis zona occludens 1 (white) in human brain microvascular endothelial cell (HBMEC) monolayers. Nucleus labelled by Hoechst is displayed in blue. Scale bar, 100 μm. **b** Western blot expression of endothelial tight junction proteins, (N-cadherin, VE-cadherin, claudin-5, and occludin) in three HBMEC replicates. Black rectangles represent the expected molecular weight ranges for the proteins. *Claud5* claudin 5, *L* *Protein ladder*, *N-cad* N-cadherin, *Occl* occluding, *VE-cad* VE-cadherin. **c** Time-dependent uptake of fluorescently labeled EVs in HBMECs analyzed by flow cytometry. DiI, 1,1′-dioctadecyl-3,3,3′,3′ tetramethylindocarbocyanine perchlorate. **d** HBMEC viability following exposure to D-mannitol (positive control) or EVs for 24 h. **e** Schematic of the transwell experiment. **f**, **g** Passage of fluorescently labeled albumin (**f**) or dextran (10 kD) (**g**) through HBMEC monolayers after three hours of incubation. HBMEC monolayers were pre-exposed to D-mannitol or EVs for 24 h. D-mannitol dose, 200 mM; EV dose, 10^4^ EVs/cell. Data are presented as mean ± SD of three (**c**, **f**, **g**) or four (**d**) replicates. Statistical analysis was performed by a two-way analysis of variance (ANOVA) test (**c**) or a one-way ANOVA test (**d**, **f**, **g**) with post-hoc pairwise comparisons calculated with a Tukey’s test. *p < 0.05; **p < 0.01; ***p < 0.001; ****p < 0.0001

Interactions between cancer cell-derived EVs and human brain microvascular endothelial cells (HBMECs) were assessed, as endothelial cells are one of the main components of the pre-metastatic brain niche [31]. HBMECs were cultured for 10 days to form monolayers (Additional file 1: Fig. S2) with tight junctions, which are characteristic of the selectively permeable brain endothelial barrier [32]. Confocal microcopy and Western blot analysis verified the expression of junctional markers, including zona occludens 1, N-cadherin, VE-cadherin, claudin-5, and occludin expression in endothelial cells (Fig. 2a, b). HBMEC monolayers with tight junctions were exposed to cancer cell-derived EVs to assess uptake and effects on cell viability and barrier integrity. Reg-EVs and Br-EVs displayed comparable time-dependent uptake rates in HBMECs (Fig. 2c) [33], and did not show any statistically significant change in cell viability (Fig. 2d). Leakage of dextran (10 kDa) and albumin (66 kDa) across HBMEC monolayers exposed to EVs was measured to evaluate monolayer integrity (Fig. 2e). D-mannitol was used as a positive control, as it is known to damage the structure of specialized endothelial barriers in vitro [34]. Exposure of HBMEC monolayers to Reg-EVs and Br-EVs caused a similar increase in both dextran (61% increase for Reg-EVs and 69% increase for Br-EVs) and albumin (47% increase for Reg-EVs and 32% increase for Br-EVs) leakage (Fig. 2f, g). In summary, both Reg-EVs and Br-EVs decreased HBMEC monolayer integrity and barrier function, which has previously been associated with EV-mediated pro-metastatic effects [9, 12, 13], confirming expected bioactivity. However, the effects of Reg-EVs and Br-EVs were indistinguishable, which is consistent with another study demonstrating that Br-EVs and Reg-EVs are taken up to a similar extent by brain endothelial cells in vivo, despite displaying different pro-metastatic capacity [9]. These results allude to the fact that non-endothelial EV interactions may be primarily responsible for the substantial differences in the capability of Reg-EVs and Br-EVs to promote breast cancer brain metastasis. Previous studies have indicated the role of astrocytes [9]; however, interactions of EVs with circulating components have been largely overlooked.

### Reg-EVs and Br-EVs bind to LDL

Pro-metastatic EVs secreted from primary tumors are released into circulation and travel long distances to reach sites of pre-metastatic niche formation [4, 10, 35–37]. Therefore, EV interactions with circulatory components may play a role in metastasis. In this study, interactions between pro-metastatic EVs and LDL were explored, as both factors play important roles in cancer progression and metastasis [17, 18]. Previous studies have revealed that non-cancerous plasma-derived EVs bind to LDL [38], suggesting that these two biological components are capable of direct contact interactions. Despite findings that separately illustrate the importance of cancer cell-derived EVs and LDL in the spread of cancer, interactions between these two factors have not yet been explored.

**Fig. 3:**
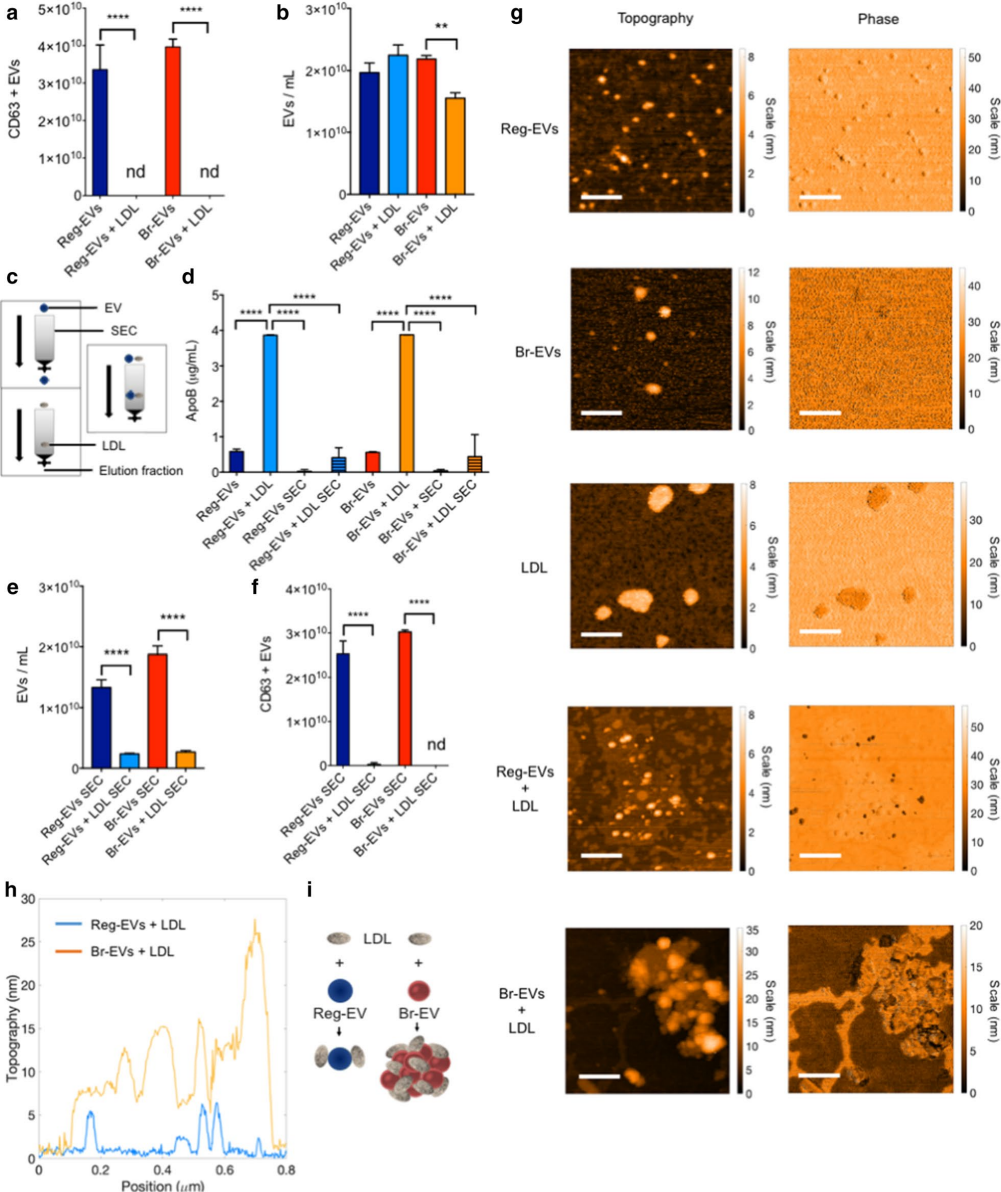
Binding of low-density lipoprotein (LDL) to breast cancer cell-derived Reg-EVs and Br-EVs. Reg EVs were derived from MDA-MB-231 cells and Br-EVs from MDA-MB-231-BrM2-831 cells. **a** Enzyme-linked immunosorbent assay (ELISA) measurements of CD63 pre and post-incubation of EVs with LDL. **b** NTA measurements of EV concentrations pre and post-incubation with LDL. **c** Schematic of the gravitational flow of EVs and LDL in a size-exclusion chromatography (SEC) column. **d** ELISA measurements of apolipoprotein B (ApoB) in EVs with and without LDL pre and post-SEC. **e** NTA measurements of EV concentrations with and without LDL post-SEC. **f** ELISA measurements of CD63-positive EVs with and without LDL post-SEC. **g** Atomic force microscopy (AFM) topography and phase images of EVs and LDL. Scale bar, 200 nm. Br-EVs + LDL display large aggregates that are not present in Reg-EVs + LDL. **h** Merged cross-sectional graph of AFM images. **i** Schematic illustrating that Br-EVs + LDL present thick and multilayered aggregates that are not present in Reg-EVs + LDL. LDL dose, 500 μg/10^10^ EVs. Data are presented as mean ± SD of three replicates. Statistical analyses were performed by one-way ANOVA (**a**, **b**, **d**, **e**, and** f**) with post-hoc pairwise comparisons calculated with a Tukey’s test. *p ≤ 0.05; **p < 0.01; ***p < 0.001; ****p < 0.0001. *nd* not detected

In this study, an enzyme-linked immunosorbent assay (ELISA) demonstrated that both cancer-derived Reg-EVs and Br-EVs bind to LDL, based on lipoprotein-mediated masking of a well-known EV surface protein [25], CD63 (Fig. 3a). Notably, the presence of LDL caused a substantial reduction in Br-EV counts compared to Reg-EVs (Fig. 3b), suggesting LDL-induced aggregation of the former. The LDL marker apolipoprotein B (ApoB) measured by ELISA was present in low amounts on Reg-EVs and Br-EVs, while detection of ApoB substantially increased upon mixing EVs with LDL (Fig. 3d). To assess whether EV-LDL interactions are maintained during gravitational flow conditions, size-exclusion chromatography (SEC) was performed. Previous studies have shown that more than 95% of lipoproteins remain entrapped in commercially available EV SEC columns (Fig. 3c) [24, 39, 40]. In accordance with previous studies, negligible amounts of ApoB could be detected when LDL was processed through SEC, as was evident from ELISA measurements (Fig. 3d). NTA measurements (Fig. 3e) and CD63 levels (Fig. 3f) revealed that EV-LDL interactions caused a statistically significant decrease in EV elution from the SEC column, suggesting that EV-LDL complexes are retained in the SEC column in the presence of gravitational flow.

To assess whether cancer cell-derived EVs display preferential interactions with LDL compared to other lipoproteins, assays were performed with high-density lipoprotein (HDL), which is also abundantly found in the blood circulation [41]. The results revealed that EVs bind to HDL; however, the presence of HDL caused a less substantial reduction of EVs in the eluted SEC fraction (Additional file 1: Fig. S3), potentially suggesting that interactions with LDL were more pronounced. Additionally, compared to Br-EVs, Reg-EVs displayed increased interactions with HDL based on pre-SEC apolipoprotein A1 (ApoA1), an HDL marker (Additional file 1: Fig. S3c), and post-SEC CD63 (Additional file 1: Fig. S3d) ELISAs.

### Br-EVs cause LDL aggregation

To assess whether breast cancer-derived Reg-EVs and Br-EVs display distinct interactions with LDL, AFM was performed. AFM images indicated a similar EV size range (Fig. 3g) as obtained by NTA and cryo-TEM. Br-EVs appeared larger than Reg-EVs with AFM (dried samples), which was not observed with the other methods (hydrated samples) and could potentially be attributed to different dehydration responses among the two EV populations. Commercial LDL displayed a diameter of 50–200 nm (Fig. 3g), which is larger than that reported for LDL in the literature (21–27 nm) [42, 43]. Notably, two different commercial sources of LDL were assessed, and both displayed a similar size. Other studies have also indicated that commercial LDL is larger than 21–27 nm [42], potentially due to slight aggregation during storage [38]. It is possible that size variations in LDL could impact experimental results, however, commercial LDL is frequently used in functional studies and is assumed to retain physiological properties [44–47]. After incubation with a fixed quantity of LDL, both EV samples appeared less monodisperse (Fig. 3g). Two distinct nanoparticle populations (differing in phase) were present in the mixed sample of Reg-EVs and LDLs, while large agglomerates characterized by a uniform phase were seen in the mixed sample of Br-EVs and LDL (Fig. 3g–i), indicating substantial aggregation. The ability of Br-EVs to induce LDL aggregation may be due to distinct lipid bilayer characteristics, as lipoproteins are thought to display dynamic interactions with cell membranes, including lipid exchange and removal [48, 49]. Removal of lipids from the LDL surface may alter hydrophobic interactions, causing lipoprotein aggregation and fusion [50]. EV lipid bilayers are derived from plasma or intracytoplasmic membranes of originating cells [51]. Cellular membranes are composed of heterogeneous classes of lipid molecules that are organized in different lipid phases (fluid and gel phases) [52], characterized by distinct biomolecular components and mechanical properties [53]. Fluorescently labeled analogs of naturally occurring lipids (i.e. lipophilic probes) can be used to assess lipid phases in synthetic and biological membranes [54]. In this study, the binding of lipophilic probes to EVs was used to evaluate potential differences in lipid phases in Reg-EVs and Br-EVs. Notably, compared to Reg-EVs, Br-EVs from breast cancer and melanoma cells demonstrated increased binding to octadecyl-based probes, i.e. amphiphilic single (octadecyl rhodamine) or double (DiI) lipophilic tails (Additional file 1: Fig. S4), which preferentially distribute in gel phases [54]. Specifically, breast cancer cell-derived Br-EVs displayed a 1.7-fold increase in DiI labeling compared to Reg-EVs (Additional file 1: Fig. S4a). The fluorescence intensity of DiI probes substantially increased after incubation with Br-EVs (Additional file 1: Fig. S4a), which is expected upon incorporation of such probes into hydrophobic compartments [55]. The accuracy of this labeling assay can be improved by SEC-mediated removal of excess unbound fluorescent probes from the solution.

After the incubation with EVs, an excess of DiI not intercalated into the EV structure may still be present in the formulations contributing to the detected fluorescence signal. Given that free DiI probes in solution processed through SEC are not eluted in the same EV fractions (Additional file 1: Fig. S4a), the samples of labeled EVs were further processed through SEC in order to eliminate the unlabeled fluorescent probes. Processing of DiI alone by SEC revealed that almost all of the dye could be removed from EV fractions (Additional file 1: Fig. S4a). Following SEC-based removal of free DiI that had not intercalated in EV membranes, the increased binding of DiI to Br-EVs compared to Reg-EVs was even more pronounced (2.2-fold difference) (Additional file 1: Fig. S4a).

Previously, we demonstrated that the addition of a SEC processing step after TFF can further increase EV purity through removal of protein-based contaminants [24]. To ensure that the substantial increase in DiI labeling of Br-EVs compared to Reg-EVs was due to EV membranes as opposed to the presence of potential protein contaminants that bind to DiI, SEC was also performed prior to labeling. In accordance with the other labeling procedures, Br-EVs labeled after SEC displayed 2.3-fold higher fluorescence intensity compared to Reg-EVs (Additional file 1: Fig. S4a), suggesting that the difference in labeling was due to the EV lipid bilayer as opposed to sample contaminants. Notably, the same trend observed with breast cancer EVs was evident in melanoma EVs, where DiI labeling was more pronounced in Br-EVs than Reg-EVs (Additional file 1: Fig. 4Sb). Similarly to DiI, octadecyl rhodamine, another probe with a single lipophilic tail that distributes in gel phases of lipid membranes [54], bound preferentially to Br-EVs compared to Reg-EVs (Additional file 1: Fig. S4c).

**Fig. 4:**
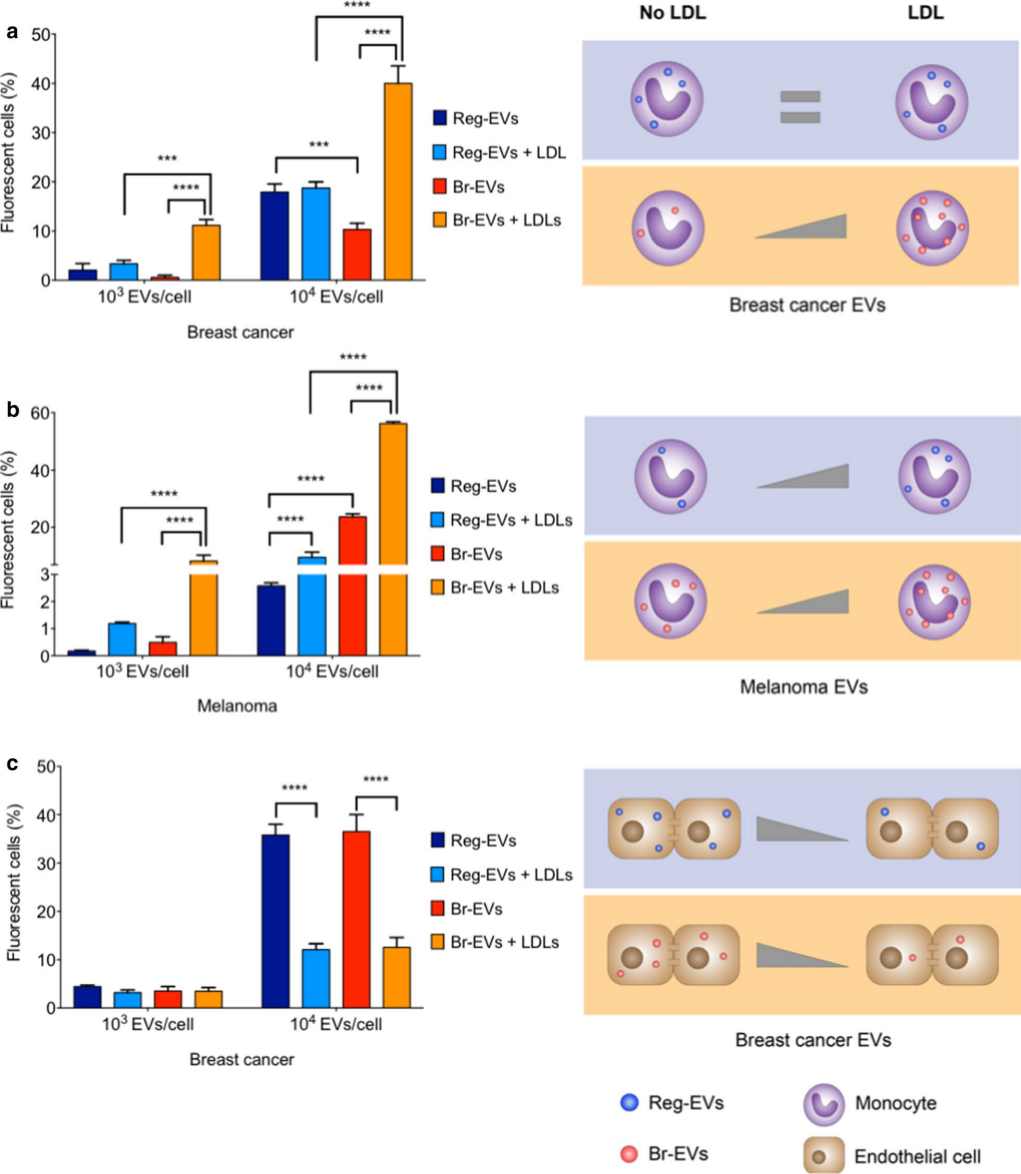
Uptake of cancer cell-derived Reg-EVs and Br-EVs in human THP-1 monocytes. Breast cancer Reg EVs were derived from MDA-MB-231 cells and breast cancer Br-EVs from MDA-MB-231-BrM2-831 cells. Melanoma Reg-EVs were derived from A375 cells and melanoma Br-EVs from M12 melanoma brain metastases cells. Cell culture media were depleted of exogenous EVs and lipoproteins. **a** Flow cytometry and corresponding schematics of THP-1 cells incubated with fluorescently labeled breast cancer EVs. **b** Flow cytometry and corresponding schematics of THP-1 cells incubated with fluorescently labeled melanoma EVs for four hours. **c** Flow cytometry and corresponding schematic of HBMECs incubated with breast cancer EVs for four hours. LDL dose, 500 μg/10^10^ EVs. Data are presented as mean ± SD of three replicates. Statistical analysis was done using two-way ANOVA with post-hoc pairwise comparisons calculated with a Tukey’s test. ***p < 0.001; ****p < 0.0001

Previously, EVs have been shown to exhibit lipid raft-like properties [56]. Accordingly, a cholesteryl ester-based lipophilic probe, cholesteryl-boron dipyrromethene (BODIPY), was used to compare potential differences in the cholesterol-rich microdomain content of Reg-EV and Br-EV membranes. Contrary to gel lipid phase probes (DiI and octadecyl rhodamine), cholesteryl-BODIPY exhibited a similar degree of binding to both Reg-EVs and Br-EVs (Additional file 1: Fig. S4d). Taken together, these results suggest that the presence of cholesterol-rich microdomains in Reg-EV and Br-EV membranes is similar, while lipid gel phases are likely to be enriched in Br-EV membranes, as indicated by the increased binding to DiI and octadecyl rhodamine. It is possible that such differences in EV lipid phases could impact interactions with LDL.

### LDL increases uptake of Br-EVs by monocytes

Aggregation of LDL has been shown to enhance its uptake by monocytes and macrophages, which may occur independently of the LDL receptor [57–59]. For example, monocytes and macrophages display scavenger receptors, such as scavenger receptor class A type 1 (SR-A1) and CD36 [50, 60] which are capable of internalizing modified and aggregated LDL. Based on the finding that Br-EVs trigger LDL aggregation, EV uptake by human THP-1 monocytes was assessed. Uptake assays were performed in cell culture medium depleted of exogenous EVs and lipoproteins. In the absence of LDL, breast cancer Br-EVs displayed reduced uptake by monocytes compared to breast cancer Reg-EVs (Fig. 4a). However, the addition of LDL caused an 18.8-fold and 3.9-fold increase in monocyte uptake of breast cancer Br-EVs at low (10^3^ EVs/cell) and high (10^4^ eV/cell) doses, respectively (Fig. 4a). Notably, the presence of LDL did not affect the uptake of breast cancer Reg-EVs by monocytes (Fig. 4a).

In the absence of LDL, melanoma Br-EVs displayed the opposite trend of breast cancer EVs, exhibiting increased uptake by monocytes compared to melanoma Reg-EVs (Fig. 4b). However, upon addition of LDL, a similar trend to that of breast cancer EVs was observed, where the presence of LDL substantially increased melanoma Br-EV uptake (16.6-fold and 2.4-fold at a dose of 10^3^ EVs/cell, and 10^4^ EVs/cell, respectively) (Fig. 4b). The presence of LDL led to a modest increase in melanoma Reg-EV uptake at the lower dose (6.9-fold) and to a comparable increase to melanoma Br-EVs at the higher dose (3.7-fold) (Fig. 4b). Taken together, these results suggest that the presence of LDL preferentially enhances Br-EV uptake compared to Reg-EV uptake by monocytes, especially in the case of breast cancer EVs. It is probable that this uptake enhancement is due to Br-EV-induced LDL association and aggregation, triggering internalization of these complexes.

To demonstrate whether LDL-induced uptake of Br-EVs was cell-type-dependent, the same studies were carried out in HBMECs, which are not known to internalize aggregated LDL. In this case, the presence of LDL did not affect HBMEC uptake of breast cancer Reg-EVs and Br-EVs at a lower dose (10^3^ EVs/cell) and substantially reduced uptake at a higher dose (10^4^ eV/cell) (Fig. 4c), displaying the opposite trend to that of monocytes. Additionally, LDL did not preferentially affect the HBMEC uptake of Br-EVs compared to Reg-EVs (Fig. 4c). These results highlight a potential association between pro-metastatic EVs, LDL, and monocytes in brain metastasis. Notably, both cancer cell-derived EVs and LDL play a key role in cancer metastasis [17, 18], whereas monocytes had previously been shown to contribute to the formation of breast cancer and melanoma metastases in animal models [61–65]. The uptake of pro-metastatic EVs in monocytes and macrophages had been shown to cause secretion of immunosuppressive factors, such as interleukin 10 (IL10), chemokine ligand 2 (CCL2), and transforming growth factor β (TGF-β), which contribute to metastatic spread [35, 66–68].

## Discussion

An important aspect of metastasis is long-distance transport of biomolecular information between primary tumors and pre-metastatic niche environments. Such transport can occur through cancer cell-derived EVs that are released into the circulation [4, 9, 10]. In fact, several studies have shown that cancer patients display higher concentrations of circulating EVs compared to healthy controls [69–74]. Multiple preclinical studies have assessed the role of EVs in metastatic spread to the central nervous system, mainly focusing on the effects of EVs on the blood–brain barrier (BBB) [9, 13, 75, 76]. In accordance with previous findings [9], this study demonstrated that Reg-EVs and Br-EVs impair the integrity of brain endothelial cells to a similar extent. Therefore, the pro-metastatic potential of Br-EVs is likely to be mediated through interactions with additional components in the pre-metastatic niche.

In addition to EVs, other biological nanoparticles in circulation, such as LDL, can promote metastasis [17, 18]. Surprisingly, interactions between lipoproteins and EVs have not previously been explored in the cancer setting, despite the pro-metastatic role and simultaneous occurrence of these nanoparticle populations in circulation and pre-metastatic niche environments. This study shows for the first time that cancer cell-derived EVs associate with LDL, and that such interactions differ based on the pro-metastatic potential of EVs. Specifically, Br-EVs cause LDL aggregation, and subsequent accelerated EV uptake by monocytes, which are known to be key components of the pre-metastatic brain niche [61–64] and have been shown to induce BBB permeability [77, 78]. Additionally, compared to breast cancer and melanoma Reg-EVs, Br-EVs displayed higher affinity for long-tailed octadecyl probes that are known to integrate in gel phases of lipid bilayers [54], indicating differences in EV membrane structure based on pro-metastatic potential.

In conclusion, this study provides the first indication that EV interactions with LDL could potentially be involved in the metastatic cascade, potentially through modulation of monocytes. Further understanding of the interplay between LDL and pro-metastatic EVs could lead to new insights into the metastatic cascade, potentially contributing to the identification of novel biomarkers and therapeutic targets to prevent cancer spread.

## Methods

### Cell lines and culture conditions

Human MDA-MB-231 breast cancer cells (HTB-26; ATCC), human MDA-MB-231-BrM2-831 breast cancer cells (provided by Dr. Joan Massagué at Memorial Sloan-Kettering Cancer Center), human A375 melanoma cells (CRL-1619; ATCC), and human M12 melanoma brain metastases cells (obtained with Mayo Clinic Institution Review Board approval under IRB#07-007623 from a patient with melanoma brain metastases) were cultured and maintained in high glucose Dulbecco’s modified eagle’s medium (DMEM) (Life Technologies) supplemented with 10% fetal bovine serum (FBS) (Sigma), 1% penicillin/streptomycin (Gemini Bioproducts), and 1% glutamine (Life Technologies) and used at passages 2–20. HBMECs (Cell System) were cultured and maintained in Complete Classic Medium Kit with Serum and Culture Boost (Cell Systems) and used at passages 4–10.THP-1 human peripheral blood monocytes (TIB-202; ATCC) were cultured and maintained in Roswell Park Memorial Institute (RPMI)-1640 medium (ATCC) supplemented with 2-mercaptoethanol (0.05 mM) and 10% FBS and used at passage 2–20. All cell lines were cultured at 37 °C in 5% CO_2_.

For EV isolation, cells were seeded in 150 mm dishes with DMEM supplemented with 10% EV-depleted FBS (Exosome-depleted FBS; System Biosciences). This FBS is also depleted of lipoproteins, as EV depletion is performed with a polyethylene glycol (PEG) 8000-based precipitation protocol [79], which has been found to precipitate lipoproteins [80–83]. The conditioned medium was collected after 48 h when the cells were 90% confluent and over 95% viable (Trypan blue).

For EV incubation studies, HBMEC were cultured in cell culture medium previously ultracentrifuged at 100,000 × *g* for four hours (Optima L100XP ultracentrifuge, Type Ti 70 rotor k factor 44, Beckman Coulter) to eliminate the majority of endogenous EVs and lipoproteins. THP-1 cells were cultured in complete medium supplemented with 10% EV/lipoprotein-depleted FBS (Exosome-depleted FBS, System Biosciences).

### EV isolation by TFF

Conditioned medium was centrifuged (800 × g; 30 min; Sotvall ST 16R centrifuge, Thermo Scientific) to discard dead cells and large cellular debris. EVs were isolated using a KrosFlo Research 2i Tangential Flow Filtration System (Spectrum Labs) as previously described [23, 24]. Briefly, cell culture medium (0.5–0.8 L) was filtered using sterile hollow fiber polyethersulfone membranes with 0.65 μm (D02-E65U-07-S; Spectrum Labs) and 500 kD (D02-S500-05-S; Spectrum Labs) molecular weight cut-off pores to remove cell debris and small biomolecules, respectively. Filters were washed with sterile phosphate buffered saline (PBS; pH 7.4; 3 × volume of the filter), prior to processing the conditioned medium. The input flow rate was 80 mL/min to keep the shear rate of the feed stream below 2000s^−1^. EVs were concentrated to approximately 50 mL and diafiltrated 6 times in a sterile cryoprotective sucrose buffer (5% sucrose, 50 mM Tris, and 2 mM MgCl; pH 7.4; 08-735B; Lonza) that has previously been used for EV studies [23, 24]. The final EV sample was concentrated to 6–9 mL ( EVs/mL) and analyzed or aliquoted in low protein binding 1.5 mL microtubes and stored at − 80 °C. To obtain a control buffer, 1 L of the sterile sucrose buffer was diafiltered 6 times using sterile hollow fiber polyethersulfone membranes with 500 KDa (D02-S500-05-S; Spectrum Labs) molecular weight cut-off pores, aliquoted into low protein binding 1.5 mL microtubes and stored at -80 °C.

### NTA

The size and concentration of isolated EVs were determined by nanoparticle tracking analysis. EVs were diluted (1:100) in sterile phosphate-buffered saline (PBS) and analyzed (1 mL) with a NanoSight LM10 (Malvern Panalytical, Malvern) (60 s measurement; 3 capture replicates).

### Cryo-TEM

A drop (∼3 µL) of RegEVs or BrEVs (10^10^/mL) was placed within a home-built controlled environment vitrification system (CEVS) on a carbon-coated perforated polymer film, supported on a 200 mesh TEM grid, mounted on a tweezer, as previously described [84]. The drop was turned into a thin film (preferably less than 300 nm) by blotting away the excess solution with a filter paper-covered metal strip. The grid was then quickly plunged into liquid ethane at its freezing point (− 183 °C). The cryo-specimens were imaged by a FEI (now Thermo Fisher Scientific, USA) Talos 200C high-resolution TEM, equipped with a Schottky field emission gun, operated at 200 kV. The specimens were maintained in the TEM at approximately − 180 °C in Gatan (USA) 626 cryo-holder. A low-dose imaging mode was used to minimize electron-beam radiation-damage, using electron exposures of less than 10 e^−^/Å^2^. Image contrast was enhanced by the TEM Volta phase-plate. Images were collected by a direct-imaging FEI Flacon III camera.

### AFM

AFM imaging was performed using an AFM Dimension V (Bruker, Billerika, MA, USA) equipped with PPP-FMR-SPL silicon tips (Nanosensors, Neuchatel, Switzerland). Briefly, EVs in sucrose buffer (50 µL) and LDL (Sigma-Aldrich, USA and Millipore Corporation, USA) were diluted 1:100 in cell and molecular ultrapure sterile water. A fixed amount of diluted EVs (50 μL) were then deposited onto freshly cleaved mica sheets (grade V-1, thickness of 0.15 mm, size of 15 mm × 15 mm), using a spin cast system model WS-650SZ-6NPP/LITE (Laurell, North Wales, PA, USA) at a speed of 800 relative centrifugal force (rcf) for 15 s. Imaging was performed using the Nanoscope Software 7.3 (Veeco, Plainview, NY, USA). The scan size ranged from 0.5 to 25 μm and the scan speed ranged from 5 μm to 10 μm per second.

### Zeta potential

The zeta potential of EVs was measured by laser Doppler micro-electrophoresis (Smoluchowski’s theory). EVs were diluted (1:100) in sterile water and analyzed with a Zetasizer Nano ZS (ZEN 3600; Malvern Panalytical).

### Western blot

Samples were mixed with sodium dodecyl sulfate (SDS)-sample buffer 6X reducing (Boston Bioproducts) and boiled (five minutes; 95 °C). MDA-MB-231 cell homogenate was used as a control for EV sample analysis. Cell homogenates were obtained by adding radioimmunoprecipitation assay (RIPA) buffer (Thermo Fisher Scientific) to the culture plates, keeping the cells on ice for five to seven minutes, scraping the cell monolayers, transferring the cells into low protein binding microtubes for centrifugation (12,000rcfforfiveminutes), and collecting the supernatant for analysis. The protein content was measured with bicinchoninic acid (BCA) assay (Thermo Fisher Scientific), and the same amount of total protein (20–50 μg protein) was loaded, electrophoresed on a polyacrylamide gel (4–12%), and analyzed by Western blot. The following antibodies were used: mouse monoclonal CD9 antibody (1:500 dilution, clone 16226D; Fisher Healthcare), mouse monoclonal anti-CD81 (1:500 dilution; clone (B11): sc-166029; Santa Cruz), rabbit polyclonal anti-Annexin V (1:500 dilution; clone ab14196; Abcam), rabbit polyclonal anti-calnexin (1:1000 dilution; clone ab10286; Abcam), mouse monoclonal anti-Claudin 5 (1:1000 dilution; clone 4C3C2; Thermo Fisher Scientific), mouse monoclonal anti-N-cadherin (1:1000 dilution; clone 3B9; Thermo Fisher Scientific), mouse monoclonal anti-VE-cadherin (1:1000 dilution; clone F-8; Santa Cruz Biotechnology), rabbit polyclonal anti-Occludin (1:1000 dilution; clone GTX114949; GeneTex, USA), anti-rabbit immunoglobulin G (IgG) secondary horseradish peroxidase (HRP)-linked antibody (1:5000; Cell Signaling Technology, USA), and anti-mouse IgG secondary HRP-linked antibody (1:5000; Thermo Fisher Scientific, USA). ECL™ Rainbow™ Marker—Full range (Amersham, USA) and BenchMark Pre-stained Protein Ladder (Invitrogen, USA) were used as protein ladders. Immunoreactive bands were identified using the Pierce ECL Western Blotting substrate (Thermo Fisher Scientific, USA) and the SuperSignal West Femto (Thermo Fisher Scientific, USA).

### Fluorescent labeling of EVs

EVs were fluorescently labeled with DiI (Thermo Fisher Scientific, USA), BODIPY (β-BODIPY™ FL C12-HPC CholEsteryl 4,4-Difluoro-5,7-Dimethyl-4-Bora-3a,4a-Diaza-s-Indacene-3-Dodecanoate) (Thermo Fisher Scientific, USA), or octadecyl rhodamine B chloride R18 (Biotium, USA). Briefly, ~ 10^10^ EVs suspended in sucrose buffer (5% sucrose, 50 mM Tris, and 2 mM MgCl), were gently mixed with DiI, BODIPY, or rhodamine (2 μL) and incubated in a water bath (37 °C) for two hours with inversion-based mixing every hour. After labeling, excess fluorescent dye was removed by SEC (see SEC section for details).

### EV incubation with LDL

DiI labeled Reg-EVs and Br-EVs were incubated in a water bath (37 °C) for two hours with human LDL (Sigma-Aldrich, USA) at a dose of 500 μg LDL/10^10^ EVs. All samples were diluted to the same final volume with sucrose buffer.

### EV incubation with HDL

DiI labeled Reg-EVs and Br-EVs were incubated in a water bath (37 °C) for two hours with human HDL (Sigma-Aldrich, USA) at a dose of 500 μg HDL/10^10^ EVs. All samples were diluted to the same final volume with sucrose buffer.

### SEC

500 µL of samples containing the same particle number (10^10^ particles/mL) was loaded on the top of a SEC column qEV original (Izon) previously washed with sucrose buffer (30 mL). Fractions 7–9 were collected and analyzed. Fluorescence intensity was measured using a 96-well plate reader (Synergy HT; Biotek) and EV concentration was measured by NTA.

### Transwell assays for dextran and albumin leakage

HBMEC cells (1.5 × 10^3^) were seeded on a porous transwell membrane (0.4 µm pores; 0.3 cm^2^ growth area; Corning 24-well polycarbonate transwell inserts) and cultured for 10 days in complete medium, replacing half of the volume with fresh medium after the first 72 h and then every 48 h. After 9 days of culture, complete medium was replaced with EV/lipoprotein-free medium and EVs (1 × 10^4^ EVs/cell), the corresponding volume of sucrose buffer (100 µL), or D-Mannitol (200 mM) was added to the lower chamber and incubated at 37 °C with 5% of CO_2_ for 24 h. The medium was then removed and replaced with fresh HBMEC EV/lipoprotein-free media. HBMEC monolayers were incubated with the same amount (20 μg) of Alexa Fluor 488 covalently labeled albumin or Alexa Fluor 488 covalently labeled Dextran 10 kDa at 37 °C with 5% CO_2_. The fluorescent molecules were carefully placed in the bottom chamber of the transwell. After three hours, the media in the upper (250 μL) and lower (850 μL) chambers of each transwell insert were collected and transferred (100 μL) into a 96-well flat-bottom plate. The fluorescent signal was read with a plate reader (Synergy HT; Biotek).

### Crystal violet staining

HBMEC monolayers were fixed (20 min) with paraformaldehyde (4%) in PBS (final pH 7.4) and then stained with Crystal violet (0.5 g/L) PBS solution (20 min). Excess staining solution was removed, and the cell monolayer was allowed to dry over night at room temperature prior to imaging.

### Immunofluorescence staining

HBMEC cells were grown on µslides 8-well Ibitreat chambers (Ibidi) for 10 days and the medium was changed every other day. The medium was removed, and cells were fixed with 4% formaldehyde (EMS) for 20 min at room temperature, washed 3 times with sterile PBS (pH 7.4), incubated with glycine (0.1 M), and permeabilized with Triton X-100 (0.02%) for five minutes at room temperature. Cells were blocked with Protein-Block reagent (Dako, X090930-2) for 30 min and stained for one hour with rabbit polyclonal anti-ZO1 (1:50 dilution; clone 617,300; Thermo Fisher Scientific), and Texas Red phalloidin (1:1000 dilution; Thermo Fisher Scientific). Cells were then washed 3 times with sterile PBS (pH 7.4) and incubated with anti-rabbit IgG AlexaFluor 488-linked antibody (green signal, 1:1000; Thermo Fisher Scientific) for one hour in the dark. Antibody dilutions were performed using antibody diluent (Dako, S302281-2). HBMECs were washed 3 times with PBS, co-stained with Hoechst for 15 min. Cell monolayers were imaged using a Zeiss LSM 880 laser scanning confocal microscope with 40 × and 63 × oil objectives. Images were processed using the Zen software (Zeiss).

### MTS (3-(4,5-dimethylthiazol-2-yl)-5-(3-carboxymethoxyphenyl)-2-(4-sulfophenyl)- 2H-tetrazolium) viability assay

HBMECs were cultured for a total of 10 days in 96-well plates (1.5 × 10^3^/well). After 9 days of culture, complete medium was replaced with EV/lipoprotein-free medium and EVs (1 × 10^9^/100 μL), the corresponding volume of sucrose buffer (100 μL), or D-mannitol (100 mM) were added. After 24 h, MTS reagent (10% volume) was added to the medium and incubated at 37 °C at 5% of CO_2_ for one hour. Absorbance at 490 nm was read with a plate reader (Synergy HT; Biotek).

### ELISA

EVs mixed with LDL prior and after SEC were analyzed with a Quantikine ELISA for ApoB (Cat. No. DAPD00, R&D Systems). The best-fit curve yielded an *R*^2^ value of 0.9869. EVs mixed with HDL prior and after SEC where analyzed with an apolipoprotein A-I (ApoA1) ELISA Kit (Cat. No. ABIN1028138, Antibodies-Online). The best-fit curve yielded an R^2^ value of 0.9788. EVs mixed with HDL or LDL prior and after SEC were analyzed with an ExoELISA-Ultra complete Kit (Cat. No. NC1242688, System Biosciences) for detection of CD63. The best-fit curve yielded an R^2^ value of 0.8689. All samples were diluted to 10^8^ EVs/well and analyzed in triplicate. Standards and samples were incubated with kit reagents according to the manufacturer’s instructions.

### Flow cytometry

DiI-labeled RegEVs and BrEVs were processed through a SEC column qEV original (Izon) to remove excess fluorophore. HBMECs (5 × 10^4^/well in 24-well plates) were grown for 9 days and incubated with two different doses of breast cancer RegEVs and BrEVs (10^3^ EVs/cell, 10^4^ EVs/cell) for three hours in EV-free media. THP-1 cells (5 × 10^4^/well in 24-well plates) were seeded in EV-free media and incubated with two different doses of purified breast cancer or melanoma RegEVs and BrEVs (10^3^ EVs/cell,  EVs/cell) for three hours. Hoechst 33258 pentahydrate (bis-benzimide) (2 μL; Invitrogen) was added to each well 15 min prior the flow cytometry analysis. THP-1 cells were collected, pelleted (800 × *g* for 5 min), resuspended in PBS (200 μL), stained with Sytox Red (Thermo Fisher Scientific) for 20 min at room temperature, and analyzed by flow cytometry (Attune NxT flow cytometer; Thermo Fisher Scientific). For each sample, a fixed number of cells (> 1 × 10^4^) was analyzed and the data from the Sytox Red-negative, Hoechst-positive, DiI-positive population was recorded (Additional file 1: Fig. S5). HBMECs were washed once with sterile PBS, trypsinized, pelleted (800 × *g* for five minutes), resuspended in PBS (200 μL), and stained/analyzed as reported for THP-1 cells (Additional file 1: Fig. S6).

### Statistical analysis

The statistical tests used to analyze the data are indicated in the figure legends. Graph construction and statistical analyses were performed using Prism 7.0a software (GraphPad).

## Supplementary information

**Additional file 1:** Supplementary information.

## Data Availability

The authors declare that the data are available in the main manuscript, Supplementary Information files, and from the corresponding authors upon reasonable request. Correspondence and requests for materials should be addressed to JW and SB.
