# Supplementary material for: Brain metastases-derived extracellular vesicles induce binding and aggregation of low-density lipoprotein
Source: J Nanobiotechnology. 2020 Nov 7;18:162. doi: 10.1186/s12951-020-00722-2 (PMC7648399; doi:10.1186/s12951-020-00722-2)
Supplement: Supplementary file 1 — Additional file 1: Supplementary information. [file 12951_2020_722_MOESM1_ESM.docx]

**SUPPLEMENTARY INFORMATION**

**Brain metastases-derived extracellular vesicles induce binding and aggregation of low-density lipoprotein**

Sara Busatto^1,2,3*^, Yubo Yang^1^, Sierra A. Walker^1^, Irina Davidovich^5^, Wan-Hsin Lin^4^, Laura Lewis-Tuffin^4^, Panagiotis Z. Anastasiadis^4^, Jann Sarkaria^6^, Yeshayahu Talmon^5^, Gregory Wurtz^7^, Joy Wolfram^1,*^

^1^Department of Biochemistry and Molecular Biology, Department of Physiology and Biomedical Engineering, Department of Transplantation, Mayo Clinic, Jacksonville, FL 32224, USA

^2^Vascular Biology Program, Boston Children’s Hospital, Boston, MA USA

^3^Department of Surgery, Boston Children’s Hospital and Harvard Medical School, Boston MA USA

^4^ Department of Cancer Biology, Mayo Clinic Comprehensive Cancer, Center, Mayo Clinic, Jacksonville, FL, USA.

^5^ Department of Chemical Engineering and the Russell Berrie Nanotechnology Institute (RBNI), Technion-Israel Institute of Technology, Haifa 3200003, Israel.

^6^ Department of Radiation Oncology, Mayo Clinic, Rochester, MN 55902, USA.

^7^ Department of Physics, University of North Florida, Jacksonville, FL 32224, USA.

*Co-corresponding authors

^
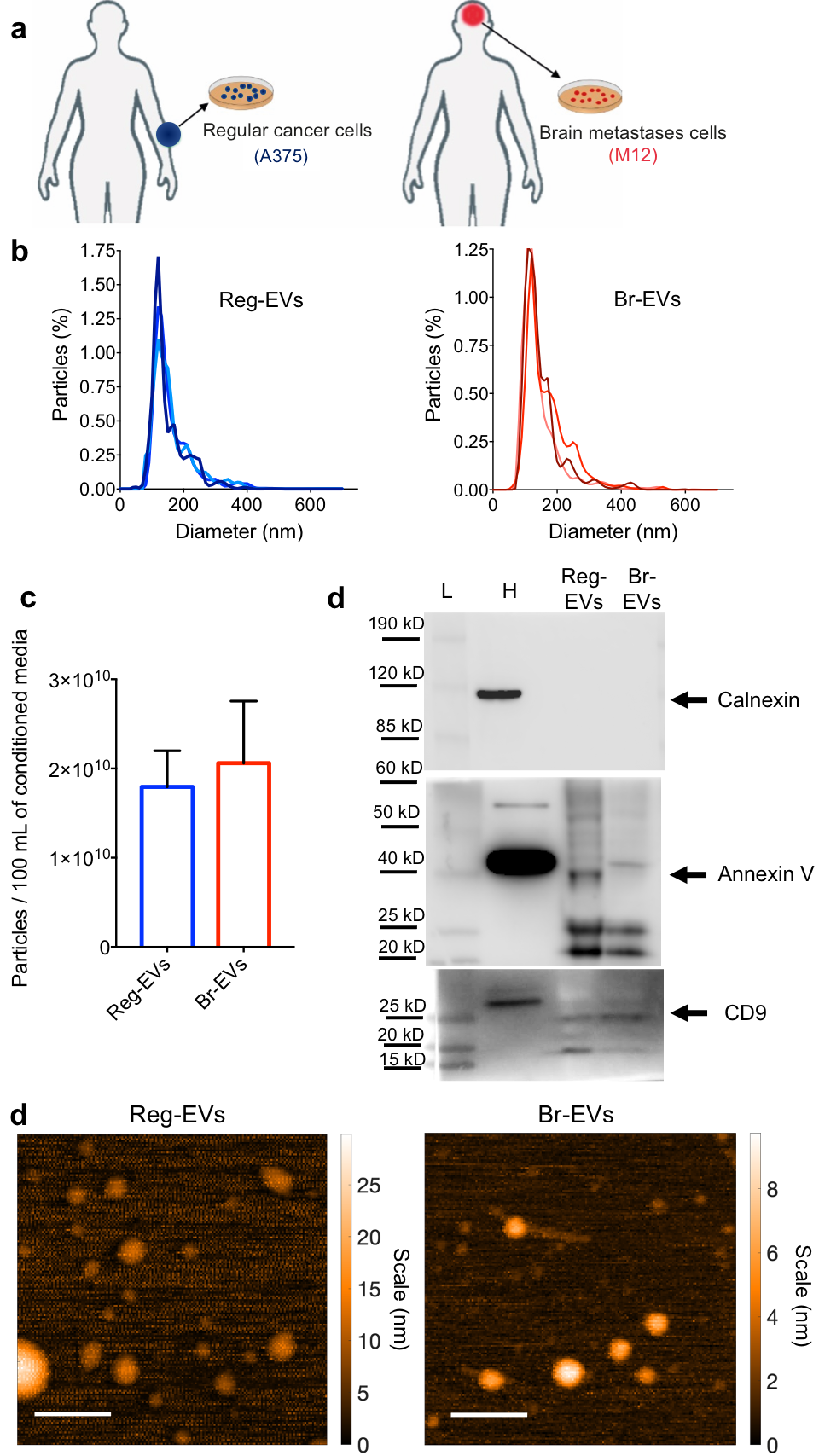
^

**Supplementary Figure 1. Characterization of extracellular vesicles (EVs) derived from regular (Reg) and brain metastases (Br) melanoma cells.** The conditioned media of regular human A375 melanoma cells and human M12 melanoma brain metastases cells was processed by tangential flow filtration to obtain Reg-EVs and Br-EVs, respectively. **a** Schematic of cell source. **b** Size distribution profiles (10 nm increments) obtained with nanoparticle tracking analysis (NTA) of three biological replicates. **c** Particles isolated from 100 mL of conditioned cell culture media. **d** Protein markers of EVs (annexin V and cluster of differentiation (CD)9) and intracellular contaminants (calnexin) obtained with Western blot. Arrows represent the expected protein band. H, cell homogenate; L, protein ladder. **e** Representative atomic force microscopy images; scale bar, 200 nm.

**
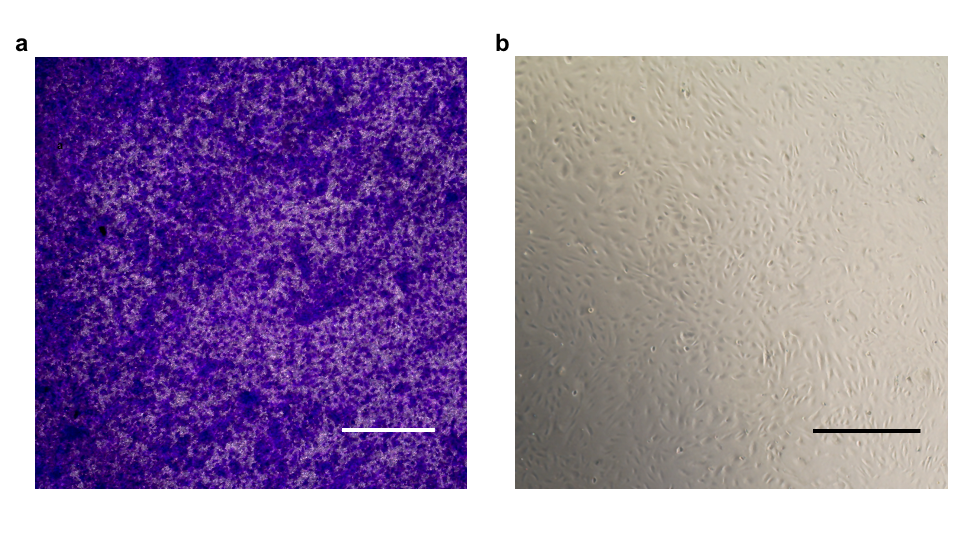
**

**Supplementary Figure 2. a** Crystal violet staining of a representative human brain microvascular endothelial cell (HBMEC) monolayer after ten days of cell culture. Scale bar, 1000 µm. **b**, Bright field image representative human brain microvascular endothelial cell (HBMEC) monolayer after ten days of cell culture. Scale bar, 500 µm.


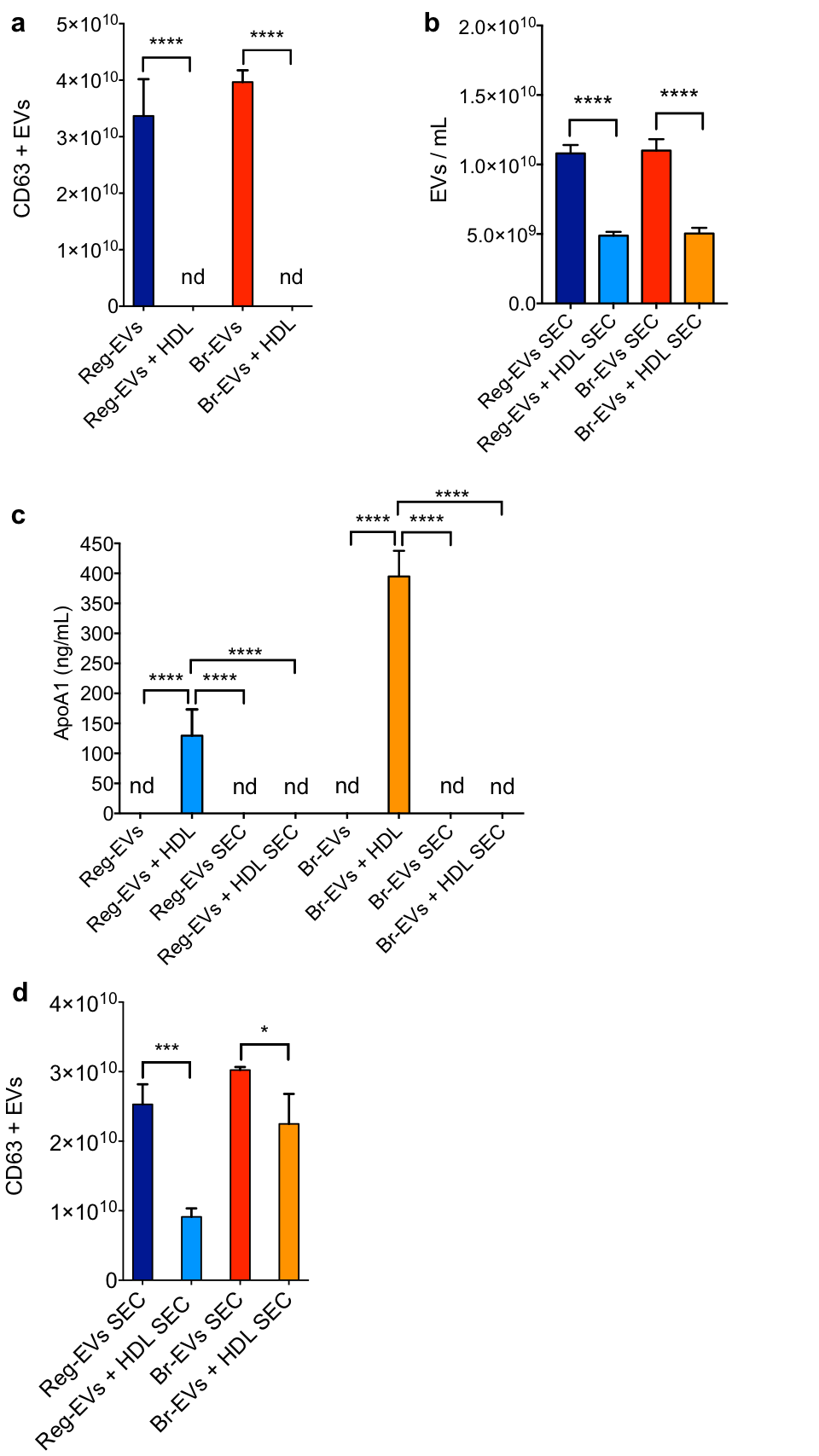


**Supplementary Figure 3. Binding of high-density lipoprotein (HDL) to breast cancer cell-derived Reg-EVs and Br-EVs.** Breast cancer Reg EVs were derived from MDA-MB-231 cells and breast cancer Br-EVs from MDA-MB-231-BrM2-831 cells. **a** Enzyme-linked immunosorbent assay (ELISA) measurements of cluster of differentiation (CD) 63 pre and post-incubation of EVs with HDL. **b** NTA measurements of EVs with and without HDL post-size-exclusion chromatography (SEC). **c** ELISA measurements of apolipoprotein A1 (ApoA1) in EV with and without HDLs pre and post-SEC. **d** ELISA measurements of CD63 in samples shown in supplementary Fig. 3a, namely EVs alone or incubated with HDL, post-size-exclusion chromatography (SEC). HDL dose, 500 μg/10^10^ EVs. Data are presented as mean ± SD of three replicates. Data shown for Reg-EVs and Br-EVs (without HDL) in **a-d** are the same as in figure 3 and are shown for comparison purposes (results in this figure and figure 3 are based on an experimental run that was performed simultaneously to provide the most accurate comparison of EV binding to LDL vs. HDL). as experiments here and in figure 3 were performed simultaneously and the same Reg-EVs and Br-EVs were used. Statistical analysis was performed by one-way analysis of variance (ANOVA) test (**a-d**) with post-hoc pairwise comparisons calculated with a Tukey’s test. *, *p* < 0.05; ***, *p* < 0.001; ****, *p* < 0.0001. nd, not detected.


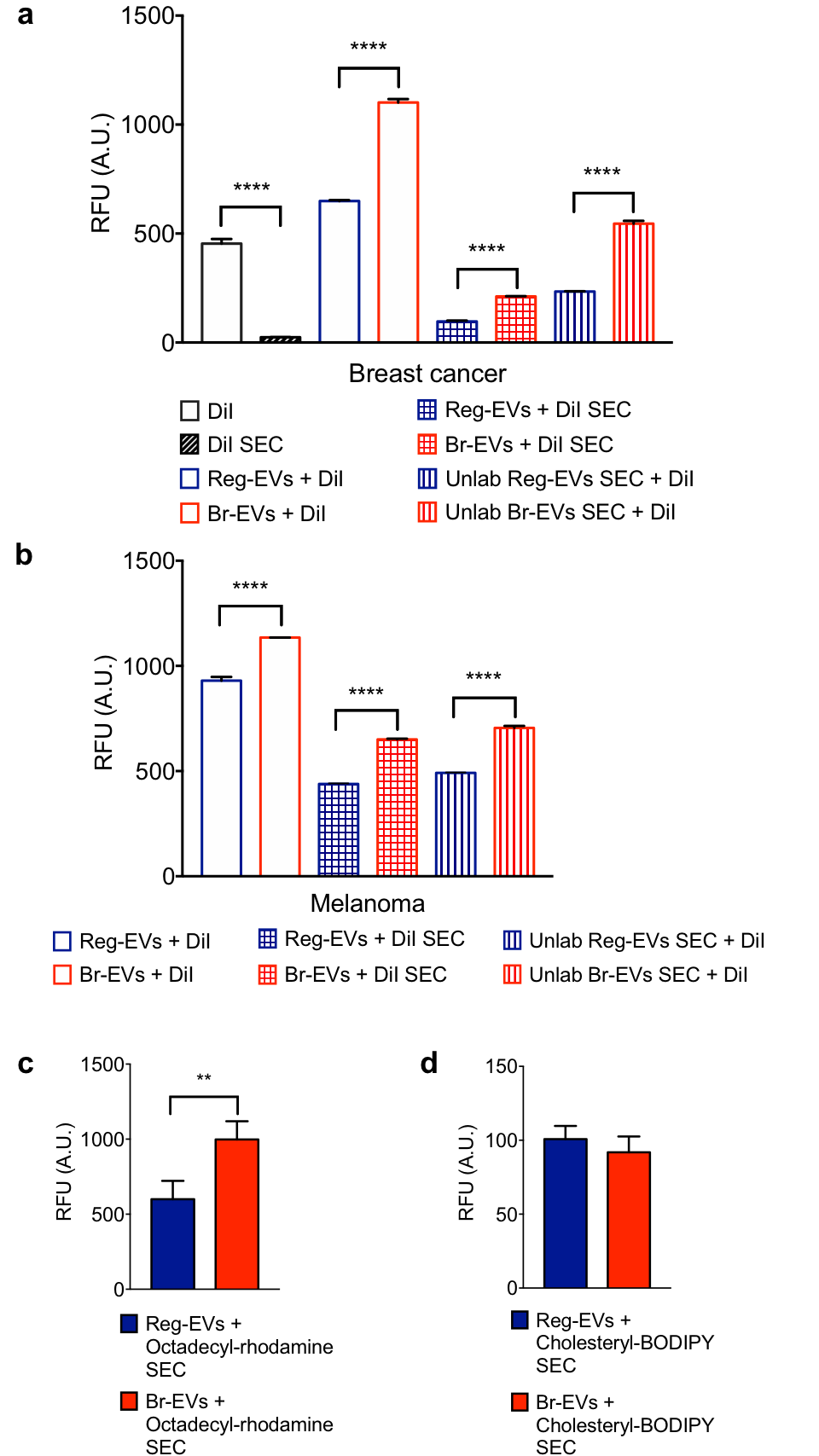


**Supplementary Figure 4.** **Labeling of cancer cell-derived Reg-EVs and Br-EVs with lipophilic probes. a** Breast cancer Reg EVs were derived from MDA-MB-231 cells and breast cancer Br-EVs from MDA-MB-231-BrM2-831 cells. Melanoma Reg-EVs were derived from A375 cells and melanoma Br-EVs from M12 melanoma brain metastases cells. Fluorescence intensity of DiI-labeled breast cancer EVs with or without SEC. **b** Fluorescence intensity of DiI-labeled melanoma EVs with or without SEC. **c, d** Fluorescence intensity of octadecyl rhodamine (**c**) or cholesteryl-boron dipyrromethene (BODIPY) (**d**)-labeled breast cancer EVs post-SEC. Data are presented as mean ± SD of three replicates. Statistical analysis was performed by one-way analysis of variance (ANOVA) test (**a-d**) with post-hoc pairwise comparisons calculated with a Tukey’s test. **, *p* < 0.01; ****, *p* < 0.0001. A.U., arbitrary units; RFUs, relative fluorescent units; unlab, unlabeled.


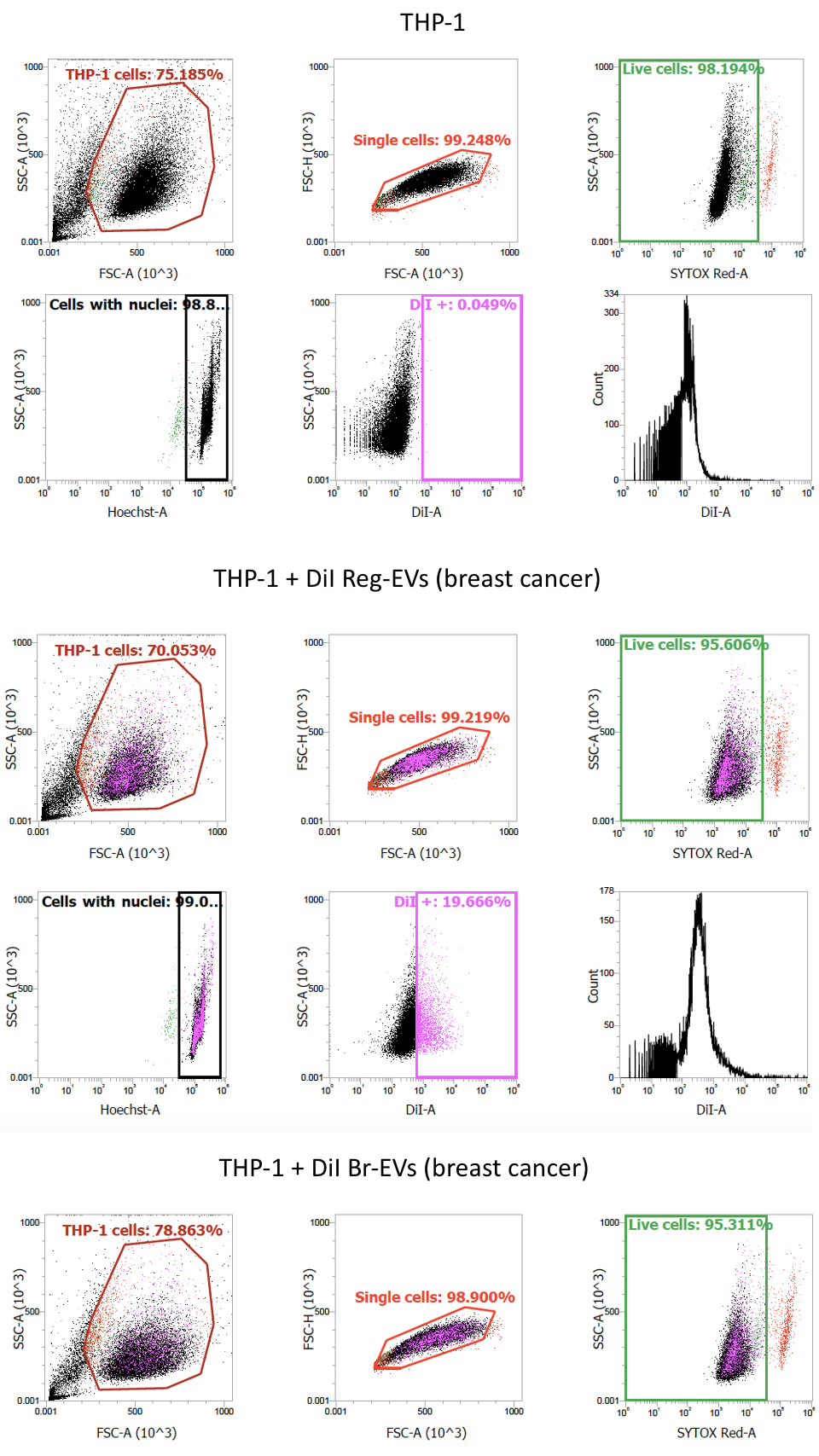


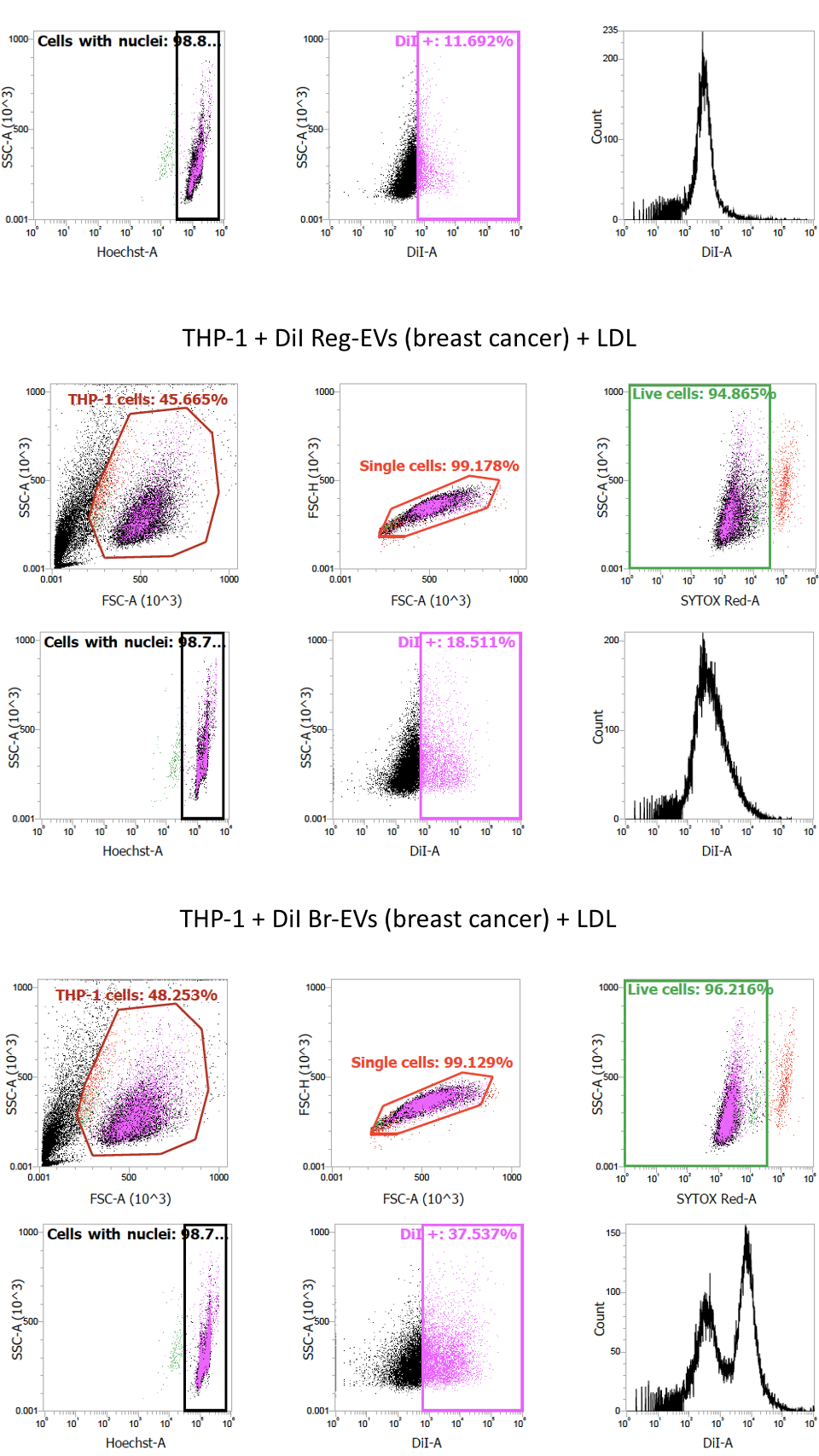


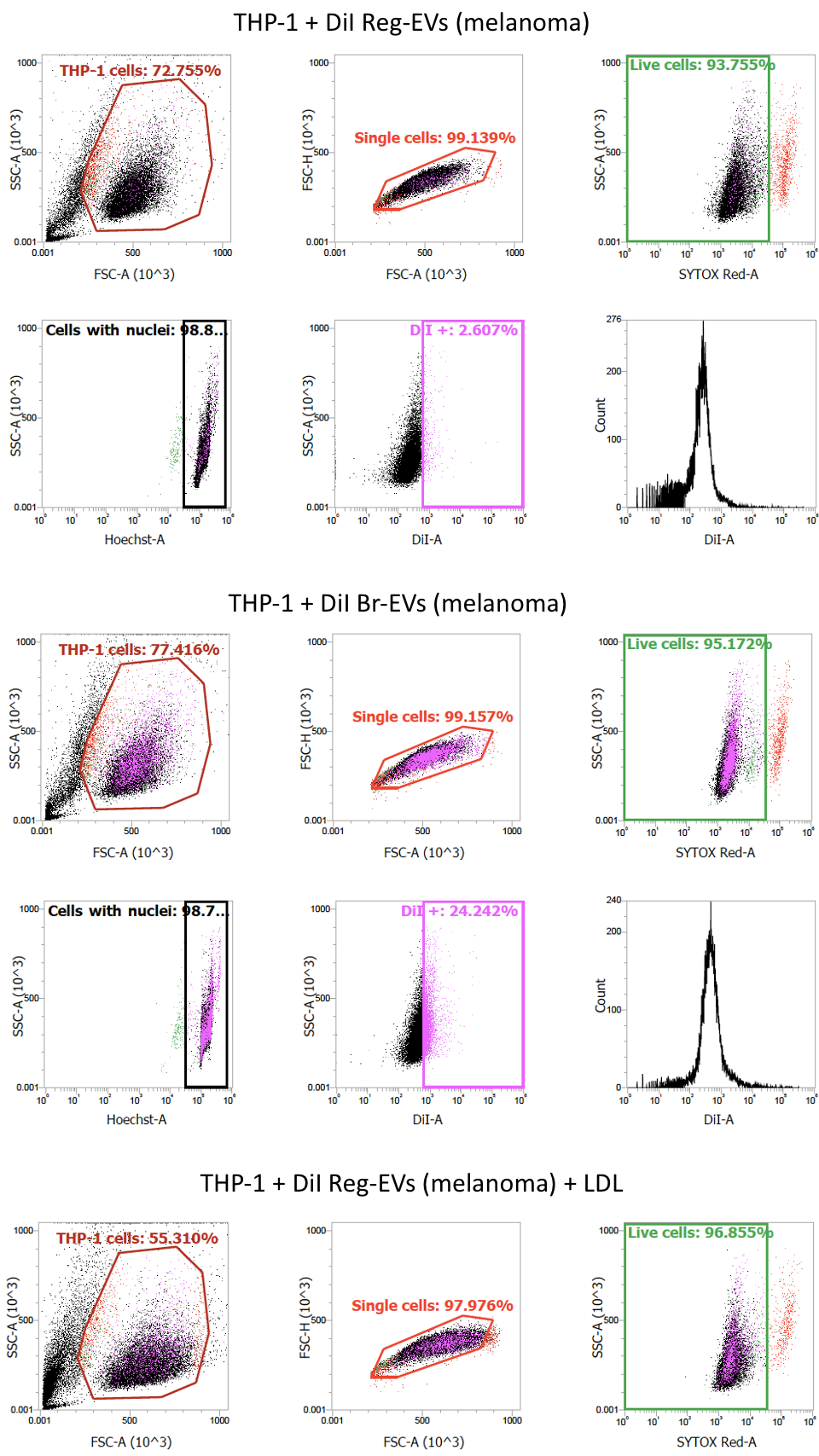


**
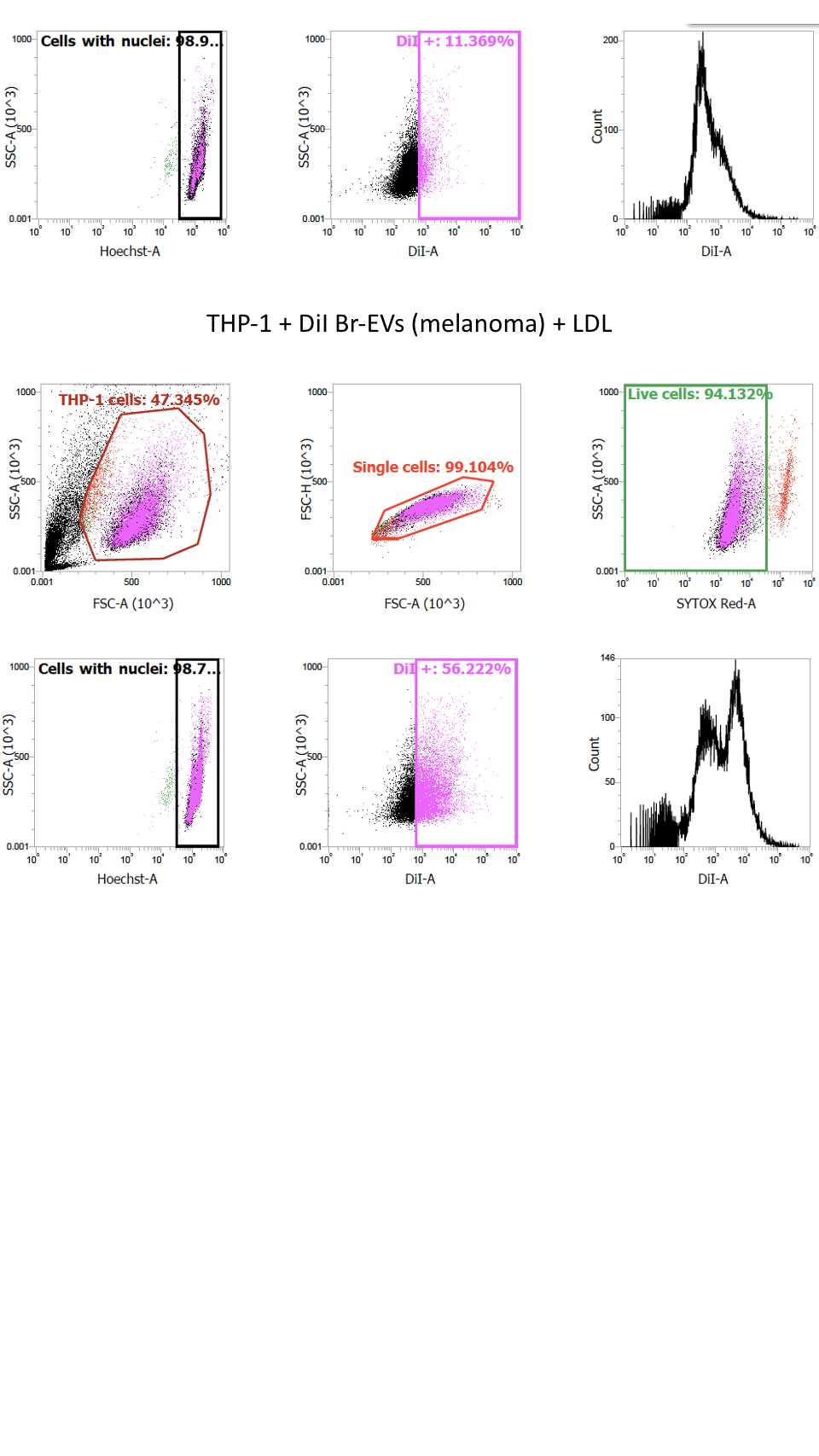
**

**Supplementary Figure 5. Flow cytometry gating strategy for uptake experiments of cancer cell-derived Reg-EVs and Br-EVs in human THP-1 monocytes.** Representative plots of THP-1 cells. A, area; DiI, 1,1'-dioctadecyl-3,3,3',3' tetramethylindocarbocyanine perchlorate; FSC, forward scatter; H, height; LDLs, low-density lipoprotein; SSC, side scatter.


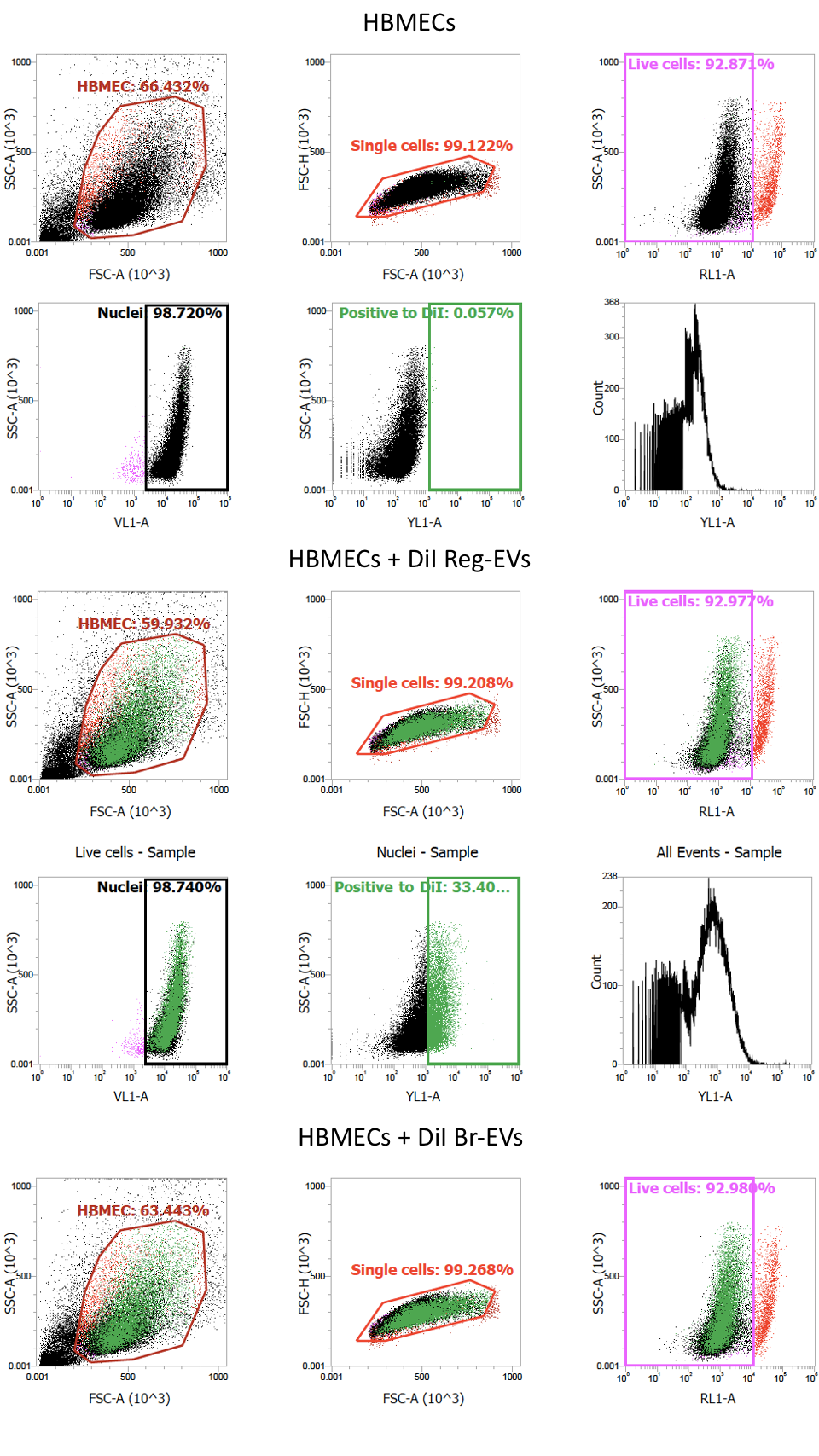


**
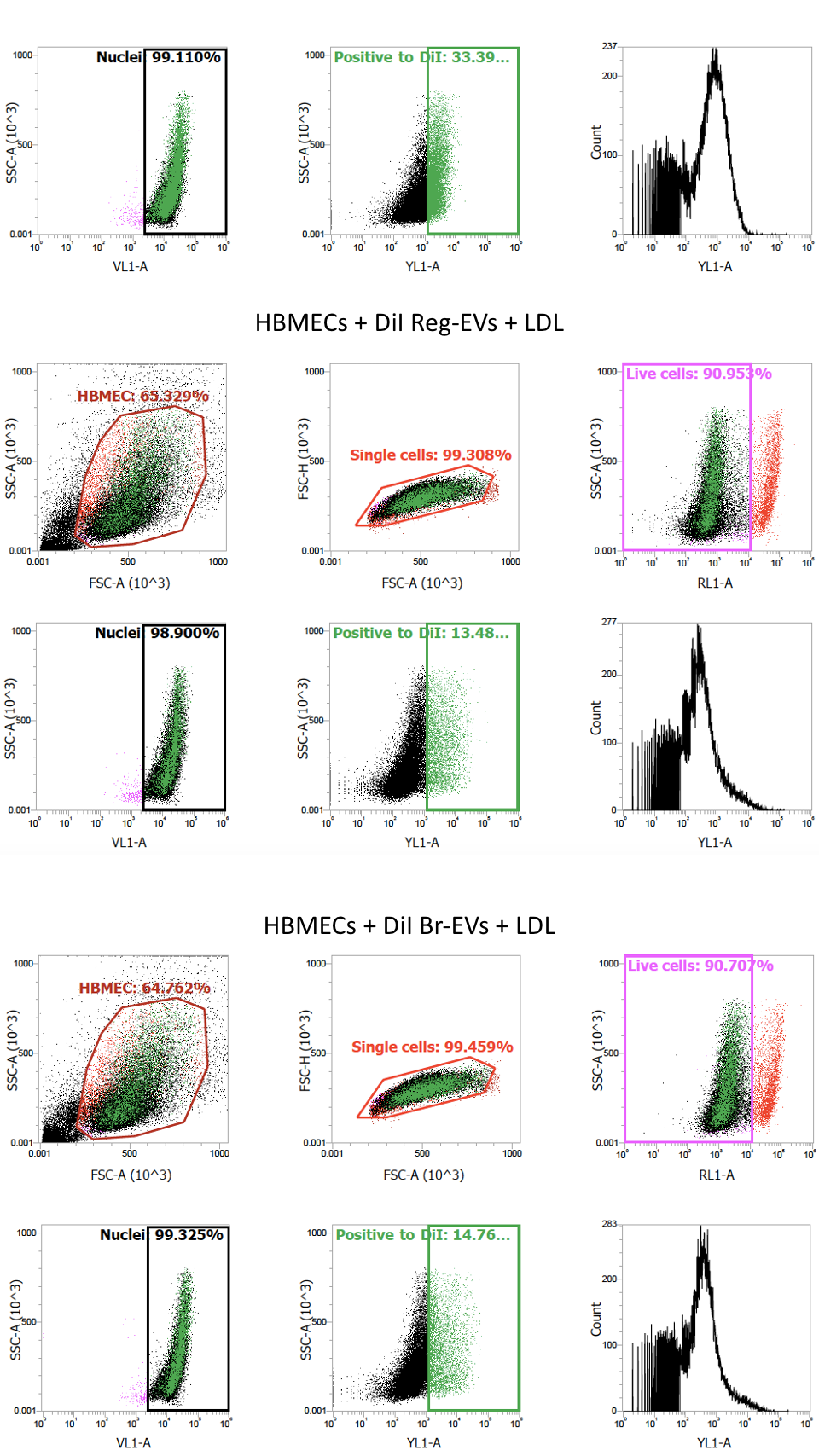
**

**Supplementary Figure 6. Flow cytometry gating strategy for uptake experiments of cancer cell-derived Reg-EVs in and Br-EVs in HBMECs.** Representative plots of HBMECs. RL1, red laser 1 (Sytox Red); VL1, violet laser 1 (Hoechst); YL1, yellow laser 1 (DiI).
